# Supplementary material for: Hyperactive Natural Killer cells in Rag2 knockout mice inhibit the development of acute myeloid leukemia
Source: Commun Biol. 2023 Dec 21;6:1294. doi: 10.1038/s42003-023-05606-3 (PMC10739813; doi:10.1038/s42003-023-05606-3)

Supplemental Information

Sugimoto E et al. **Hyperactive Natural Killer cells in *Rag2* knockout mice inhibit the development of acute myeloid leukemia**

Supplementary Table 1, 2, 3. 1-2

Supplemental Figure 1-4. 3-6

Supplementary Table 1. Characters of mouse myeloid leukemia cells used in this study.

Tumor name	Oncogene	Strain	Recipient mice	Leukemia development
MLL-AF9	MLL-AF9	C57BL/6	non-irradiation	faster in WT than *Rag2* $^{-/-}$
RUNX1-ETO9a	RUNX1-ETO9a	C57BL/6	non-irradiation	faster in WT than *Rag2* $^{-/-}$
cRAM	RUNX1-S291fsX300 and ASXL1-p.E635RfsX15 KI	C57BL/6	sublethal irradiatioin	faster in *Rag2* $^{-/-}$ than WT
cSAM-original	SETBP1-D868N and ASXL1-E635RfsX15	C57BL/6	sublethal irradiatioin	faster in *Rag2* $^{-/-}$ than WT
cSAM-2020	SETBP1-D868N and ASXL1-E635RfsX15	C57BL/6	non-irradiation	faster in WT than *Rag2* $^{-/-}$

WT: Wild-type mice, *Rag2* $^{-/-}$: Rag2 knockout mice

Supplementary Table 2. Lists of antibodies used in this study.

Antibody	Clone	SOURCE	IDNENTIFIER	Dillution
APC-conjugated anti-mouse NK1.1	PK136	BioLegend	Cat#108710	1:400
APC anti-mouse CD107a (LAMP-1) Antibody	1D4B	BioLegend	Cat#121614	1:400, 1:100
APC anti-mouse IFN-γ Antibody	XMG1.2	BioLegend	Cat#505810	1:100
APC anti-mouse CD274 (B7-H1, PD-L1) Antibody	10F.9G2	BioLegend	Cat#124311	1:400
APC/Cy7-conjugated anti-mouse CD45.1	A20	BioLegend	Cat#110716	1:400
PE-conjugated anti-mouse NK1.1	PK136	eBioscience	Cat#12-5941-83	1:400
PE-conjugated anti-mouse CD3e	145-2C11	eBioscience	Cat#12-0031-85	1:400
PE Rat Anti-Mouse CD47	miap301	BD Biosciences	Cat#563585	1:400
PE Rat Anti-Mouse Ly-6A/E (Sca1)	E13-161.7	BD Pharmingen	Cat#553336	1:400
PE anti-mouse CD155 (PVR) Antibody	TX56	BioLegend	Cat#131507	1:400
PE Hamster Anti-Mouse CD54 (ICAM-1)	3E2	BD Pharmingen	Cat#553253	1:400
PE Mouse Anti-Mouse H-2Kb	AF6-88.5	BD Biosciences	Cat#553570	1:400
PE Mouse Anti-Mouse Qa-1(b)	6A8_6F10_1A6	BD Pharmingen	Cat#566640	1:400
PE/Cyanine7 anti-mouse CD117 (c-Kit) Antibody	2B8	BioLegend	Cat#105814	1:400
PE/Cy7-conjugated anti-mouse CD3e	145-2C11	BioLegend	Cat#552774	1:400
PE/Cyanine7 anti-mouse CD69 Antibody	H1.2F3	BioLegend	Cat#104511	1:400
CD49b (Integrin alpha 2) Monoclonal Antibody (DX5), PE	DX5	eBioscience	Cat#12-5971-83	1:400
CD48 Monoclonal Antibody (HM48-1), PE,	HM48-1	eBioscience	Cat#12-0481-81	1:400
CD273 (B7-DC) Monoclonal Antibody (TY25), PE	TY25	eBioscience	Cat#12-5986-81	1:400
Mouse Rae-1 Pan Specific PE-conjugated Antibody	186107	R&D Systems	Cat#FAB17582P	1:400
Mouse ULBP-1/MULT-1 APC-conjugated Antibody	237104	R&D Systems	Cat#FAB2588A	1:400
Armenian Hamster IgG Isotype Control (eBio299Arm), PE	eBio299Am	eBioscience	Cat#12-4888-83	1:400
PE Mouse IgG1, κ Isotype Control	X40	BD Biosciences	Cat#349043	1:400
Rat IgG2a kappa Isotype Control (eBR2a), PE	eBM2a	eBioscience	Cat#12-4321-83	1:400
Rat IgG2b kappa Isotype Control (eB149/10H5), PE	eB149/10H5	eBioscience	Cat#12-4031-83	1:400
Rat IgG2a kappa Isotype Control (eBR2a), APC	eBR2a	eBioscience	Cat#17-4321-81	1:400
Rat IgG2b kappa Isotype Control (eB149/10H5), APC	eB149/10H5	eBioscience	Cat#17-4031-82	1:400
NK1.1/CD161 (E6Y9G) Rabbit mAb	E6Y9G	Cell Signaling Technology	Cat#39197	1:400

Supplementary Table 3. Actual p-values used in the Figures.

Figure No.		comparison	p value	sign	test
Figure1	b	WT-Rag2-/-	0.0306	*	Log-rank
Figure1	b	WT_NSG	0.0074	**	Log-rank
Figure1	c	GFP+ cells number	0.0022	**	Mann Whitney test
Figure1	d	spleen (g)	0.0001	***	Student's t test
Figure1	d	spleen (cm)	<0.0001	****	Student's t test
Figure1	e	WT-DS_Rag-DS	0.0223	*	Log-rank
Figure2	d	Exp.1	0.0036	**	Student's t test
Figure2	d	Exp.2	<0.0001	****	Student's t test
Figure3	d	Rag2_NT-sg1	<0.0001	****	Tukey's multiple comparison test
Figure3	d	Rag2_NT-sg2	<0.0001	****	Tukey's multiple comparison test
Figure3	d	WT_NT-sg1	0.0022	**	Mann Whitney test
Figure3	d	NSG_NT-sg1	0.3095	not significant	Mann Whitney test
Figure3	f	NT-sg1	0.0187	*	Dunnett's multiple comparison test
Figure3	f	NT-sg2	0.0007	***	Dunnett's multiple comparison test
Figure4	a	PB	0.0022	**	Mann Whitney test
Figure4	a	BM	0.0040	**	Mann Whitney test
Figure4	a	Spleen	0.0040	**	Mann Whitney test
Figure4	b	CD107a	0.0040	**	Mann Whitney test
Figure4	b	CD69	0.0061	**	Mann Whitney test
Figure4	b	Sca-1	0.0040	**	Mann Whitney test
Figure4	c	NK ratio=1	0.0004	***	Student's t test
Figure4	c	NK ratio=3	0.0053	**	Student's t test
Figure4	c	NK ratio=10	0.0004	***	Student's t test
Figure4	c	NK ratio=30	0.0194	*	Student's t test
Figure4	d	CD107a_WT_(-)-YAC-1	<0.0001	****	Tukey's multiple comparison test
Figure4	d	CD107a_WT_YAC-1 - Rag2_YAC-1	<0.0001	****	Tukey's multiple comparison test
Figure4	d	CD107a_Rag2_(-)-YAC-1	<0.0001	****	Tukey's multiple comparison test
Figure4	e	IFNr_WT_(-) - (+)	<0.0001	****	Tukey's multiple comparison test
Figure4	e	IFNr_WT_(+) - Rag2_(+)	0.0117	*	Tukey's multiple comparison test
Figure4	e	IFNr_Rag2_(-) - (+)	<0.0001	****	Tukey's multiple comparison test
Figure4	h	Rag_IgG-anti-NK1.1	0.0006	***	Log-rank
Figure5	a	9a_WT_DS-Rag2_DS	0.0050	**	Log-rank
Figure5	a	GFP (%)_WT-Rag2	0.0022	**	Mann Whitney test
Figure5	b	cRAM_WT-Rag2	0.0118	*	Log-rank
Figure5	b	GFP (%)_WT-Rag2	0.0390	*	Mann Whitney test
Figure5	c	survival_WT/vehicle-Rag2/vehicle	0.0443	*	Log-rank
Figure5	c	survival_WT/DS-Rag2/DS	0.0239	*	Log-rank
Figure5	c	GFP (%)_WT-Rag2	0.0043	**	Mann Whitney test
Figure5	d	survival_cSAM-2020	0.0010	**	Log-rank
Figure5	d	GFP (%)_WT-Rag2	0.0022	**	Mann Whitney test
Figure6	b	survival_MLL-AF9-2021	0.0061	**	Log-rank
Figure6	b	GFP (%)_WT-Rag2	0.0003	***	Mann Whitney test
Figure6	c	survival_MLL-AF9-2022	0.1447	not significant	Log-rank
Figure6	c	GFP (%)_WT-Rag2	0.0111	*	Mann Whitney test
Supplemental Figure2	a	PB_WT-Rag2-/-	0.0022	**	Mann Whitney test
Supplemental Figure2	a	BM_WT-Rag2-/-	0.0040	**	Mann Whitney test
Supplemental Figure2	a	Spleen_WT-RAg2-/-	0.0040	**	Mann Whitney test

Supplemental Figure1

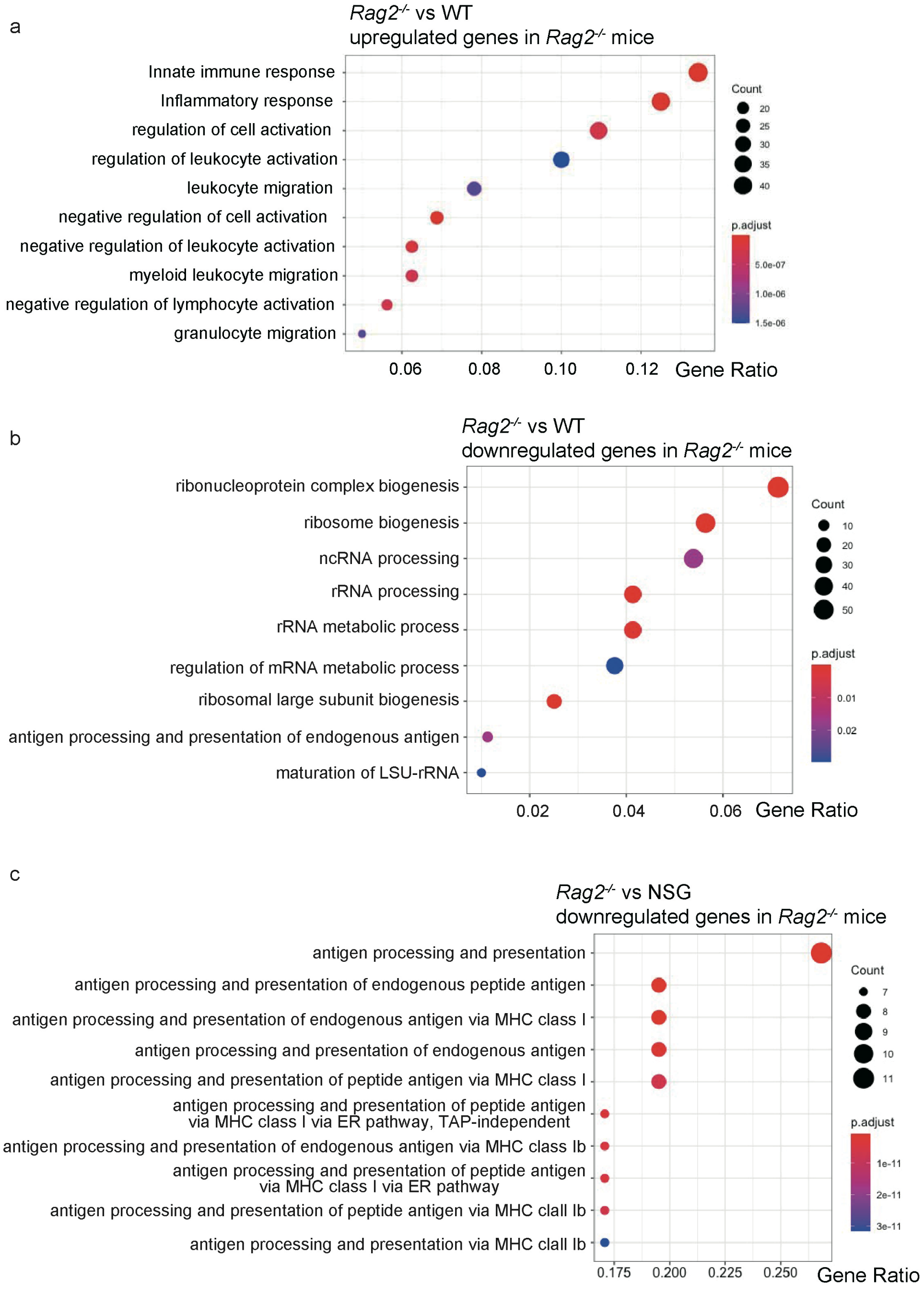

(Relates to Figure 2b)

a. GO analysis of upregulated genes in MLL-AF9 AML cells collected from *Rag2*$^{-/-}$ mice compared with those from wild-type (WT) C57BL/6 mice. Inflammation- and immune-related genes were upregulated in MLL-AF9 cells collected from *Rag2*$^{-/-}$ mice. **b**, **c**. GO analysis of downregulated genes in MLL-AF9 AML cells collected from *Rag2*$^{-/-}$ mice compared with those from WT (**b**) or NSG (**c**) mice.

Supplemental Figure 2

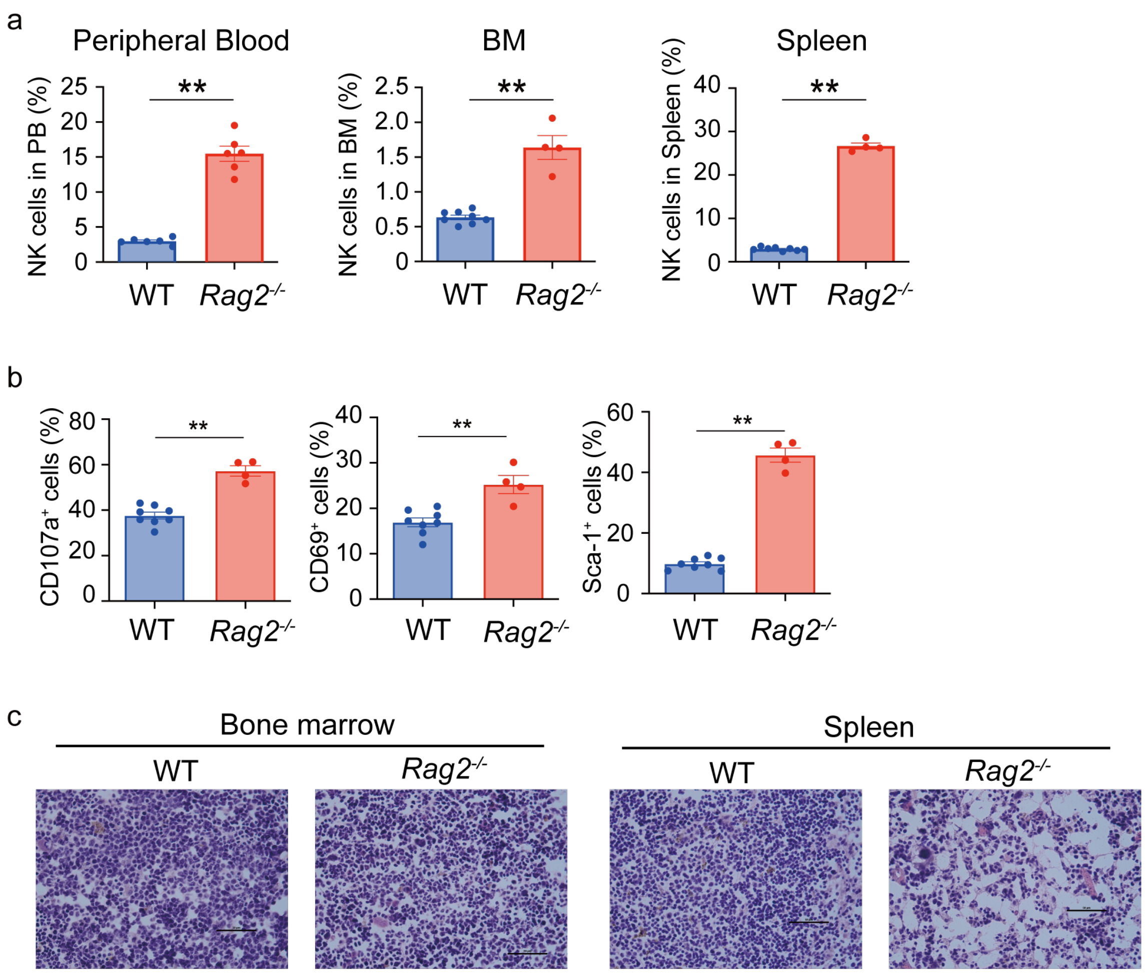

(Relates to Figure 4)

a. Frequency of NK cells in peripheral blood (PB), born marrow (BM, both femurs and tibias) and spleen. Data are means ± s.e.m. (PB n=6 per group, BM and spleen WT: n=8, *Rag2*$^{-/-}$: n=4, male mice). **P<0.01; two-tailed Mann Whitney test. See also Figure 4a.

b. Expression of NK cell activation markers (CD107a, CD69, Sca-1) in BM-derived NK cells of WT and *Rag2*$^{-/-}$ mice. Frequency of CD107a^{+}, CD69^{+}, Sca-1^{+} cells are shown as means ± s.e.m. (WT: n=8, *Rag2*$^{-/-}$: n=4). **P<0.01; two-tailed Mann Whitney test. See also Figure 4b.

c. WT and *Rag2*$^{-/-}$ mice (male) were transplanted with MLL-AF9 cells and were sacrificed 11 days after transplantation to resect tibiae and spleens. Fixed bone marrow and spleen sections from WT and *Rag2*$^{-/-}$ mice were stained with hematoxylin and eosin (H&E). Bars, 50 µm. See also Figure 4f.

Supplemental Figure 3

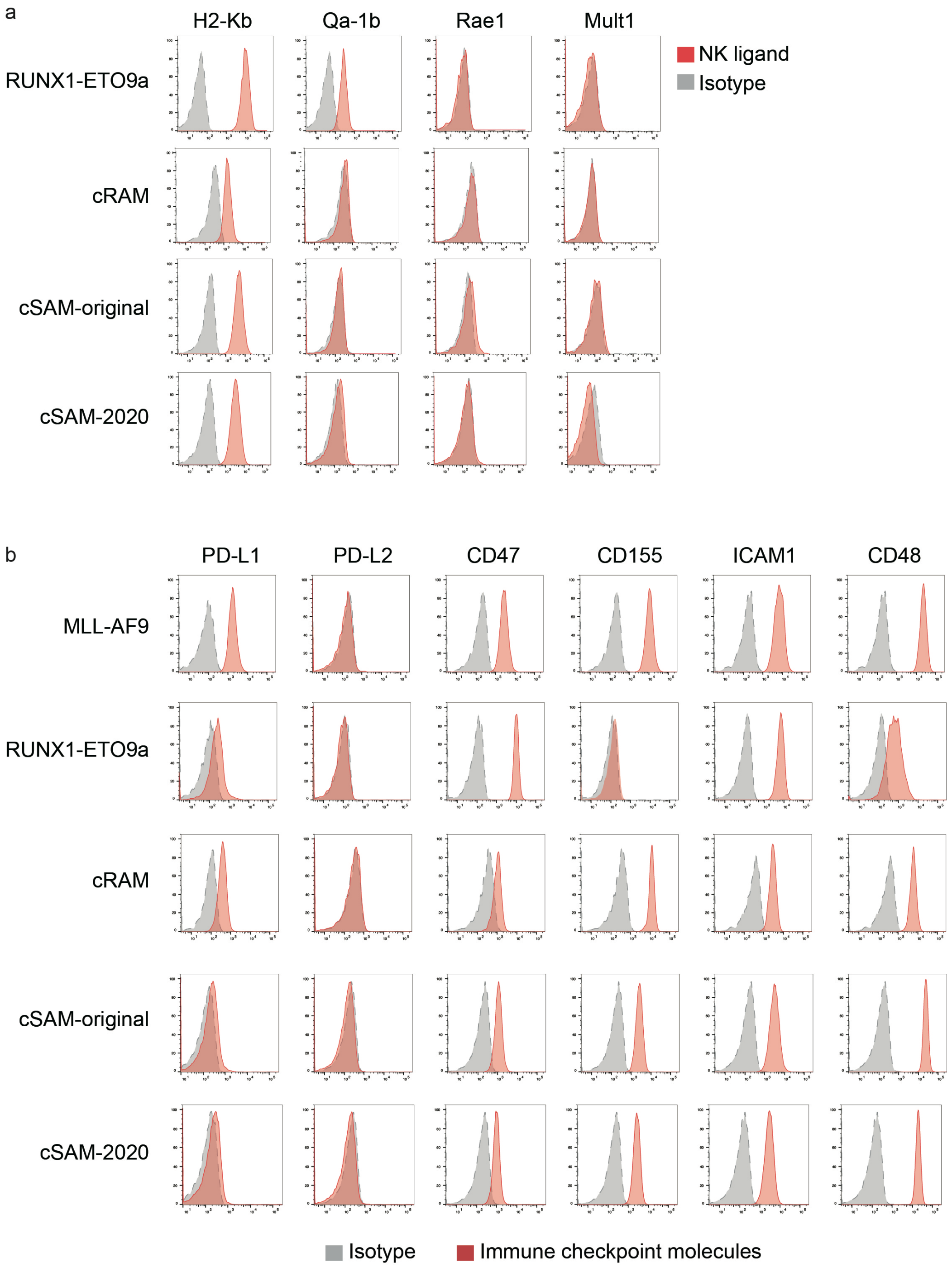

a. Flow cytometric analysis of MHC Class I molecules (H2-Kb and Qa-1b) and NKG2D ligands (Rae1 and Mult1) in myeloid neoplasms. Representative histograms are shown. **b**. Flow cytometric analysis of immune checkpoint molecules (PD-L1, PD-L2, CD47, CD155, ICAM1, CD48) in myeloid neoplasms. Representative histograms are shown.

Supplemental Figure 4: Gating strategy for FACS data

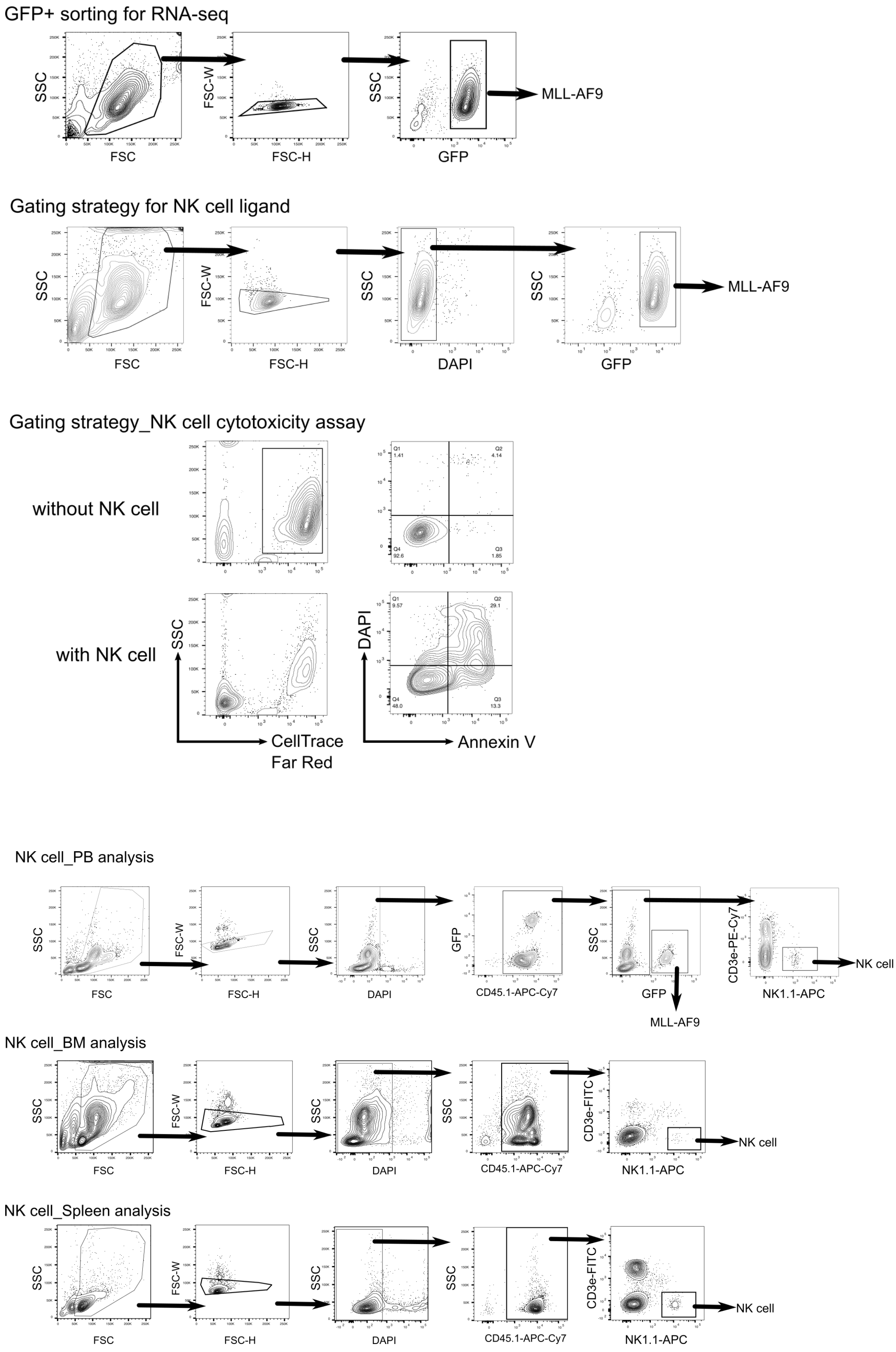

Supplement: Supplementary file 2 — Supplemental Information [file 42003_2023_5606_MOESM2_ESM.pdf]
