# Supplementary material for: Hyperactive Natural Killer cells in Rag2 knockout mice inhibit the development of acute myeloid leukemia
Source: Commun Biol. 2023 Dec 21;6:1294. doi: 10.1038/s42003-023-05606-3 (PMC10739813; doi:10.1038/s42003-023-05606-3)
Supplement: Supplementary file 3 — Description of Additional Supplementary Data [file 42003_2023_5606_MOESM3_ESM.docx]

**Description of Additional Supplementary Files**

**File name:** Supplemental Table 1

**Description:** DEG lists in MLL-AF9 cells collected from WT, Rag2^-/-^ or NSG mice.

**File name:** Supplemental Table 3

**Description:** DEG lists in MLL-AF9-2022 compared to MLL-AF9-2021.

**File name:** Supplementary Data 1

**Description:** The source data behind the graphs in the paper.
